# Maternal age and the rising incidence of hypertensive disorders of pregnancy: A comprehensive analysis of national claims data from Japan

**DOI:** 10.1371/journal.pone.0319177

**Published:** 2025-02-20

**Authors:** Naomi Maeda, Masayuki Koyama, Shintaro Takatsuka, Keisuke Oyatani, Nobuaki Himuro, Tasuku Mariya, Yoshika Kuno, Shiro Hinotsu, Tsuyoshi Saito, Hirofumi Ohnishi

**Affiliations:** 1 Department of Nursing, Sapporo Medical University School of Health Sciences, Sapporo, Japan; 2 Department of Public Health, Sapporo Medical University School of Medicine, Sapporo, Japan; 3 Department of Cardiovascular, Renal and Metabolic Medicine, Sapporo Medical University School of Medicine, Sapporo, Japan; 4 Center of Medical Education, Sapporo Medical University School of Medicine, Sapporo, Japan; 5 Department of Pediatrics, Sapporo Medical University School of Medicine, Sapporo, Japan; 6 Department of Obstetrics and Gynecology, Sapporo Medical University School of Medicine, Sapporo, Japan; 7 Biostatistics and Data Management, Sapporo Medical University School of Medicine, Sapporo, Japan; University of South Africa - Florida Campus: University of South Africa - Science Campus, SOUTH AFRICA

## Abstract

**Background:**

Hypertensive disorders of pregnancy (HDP) significantly increase the risk of developing hypertension and cardiovascular disease (CVD) later in life and are a major cause of maternal mortality. However, little is known about the nationwide, long-term, all-inclusive status of HDP.

**Objective:**

To estimate the incidence of HDP from 2011 to 2019 in Hokkaido, Japan, with a focus on age groups.

**Methods:**

Using National Database (NDB) insurance medical data, a retrospective analysis was conducted. Due to the absence of direct pregnancy data, birth numbers were used as a surrogate for the number of pregnant women to calculate the incidence of HDP.

**Results:**

The average incidence rate of HDP over 9 years was 6.37%. The incidence rate was lowest among women aged 25–29 years, at 5.58% (95% confidence interval [CI]: 5.43–5.73), and increased with age, peaking at 10.58% (95% CI: 10.10–11.09) among women over 40 years. Notably, the incidence rate for women under 20 years of age was 6.70% (95% CI: 5.97–7.51), which was higher than that for women in their 20s. A mean annual increase of 0.25% in age-adjusted incidence was observed during this period, which was statistically significant (R² =  0.87, p <  0.01).

**Conclusion:**

This study reveals that the risk of developing HDP is associated with both older childbearing and younger pregnancies and follows a J-curve, suggesting that factors other than maternal aging also contribute to the increased incidence of HDP and that further research on risk factors for HDP, which is on the rise worldwide, is urgently needed.

## Introduction

Hypertensive disorders of pregnancy (HDP), which cause hypertension (blood pressure ≥  140/90 mmHg) during pregnancy, adversely affects mothers and children during the perinatal period and is one of the major causes of maternal death [1–3]. Furthermore, women affected by HDP are at high risk of developing hypertension [4–8] and cardiovascular disease (CVD) [8–13]. For long-term follow-up, it is necessary to properly understand the actual status of HDP patients.

Several studies on the incidence and prevalence of HDP in patients have been reported. The reported prevalence of HDP among pregnant women varies from 5–10% of pregnancies to approximately 5.2–8.2%, but most of these results are based on limited populations at limited time points [14,15]. According to the results of the analysis using data from 1990–2019 for 204 countries around the world, the number of HDP cases is increasing worldwide; in all countries except those with lower sociodemographic indices and human development indices, the age-standardized incidence rate has been reported to be decreasing [16]. However, the age-standardized incidence rate is a value for 100,000 people and is not calculated as a percentage of pregnant women.

One of the risk factors for HDP is advanced maternal age, and the number of patients is estimated to increase in developed countries due to the recent increase in childbearing age [17–19]. Japan is no exception, with the average age of first childbirth consistently increasing over the past decade, reaching 30.9 years in 2021, placing it among the highest average ages in developed countries [20,21].

Taken together, these findings indicate that HDP patients are at increased risk of developing CVD in the future. However, there are not enough reports providing long-term follow-up data on HDP patients at the regional level or detailed incidence rates by age group.

Here, we aim to determine all annual trends in the number of HDP patients by age group and morbidity in the Hokkaido region, the largest island in Japan, using the National Database of Health Insurance Claims and Specific Health Checkups of Japan (NDB).

## Methods

### Data source

This was a retrospective descriptive study using receipt information from the NDB, a Japanese health insurance database. The NDB is the largest database of insured people in Japan; it was created and operated by the Japanese Ministry of Health, Labor and Welfare (MHLW) in 2009 and has been available for research since 2011. Japan has had a universal health insurance system since 1961, and all citizens except welfare recipients are covered by health insurance [22]; as of 2019, welfare recipients accounted for 1.6% of the population and less than 1% of women of reproductive age [23]. In addition, only electronic data are stored in the NDB, and the electronic receipt rate was reported to be 98.3% for hospitals and clinics as of November 2020. As a result, 25 billion cases of receipt data (2 billion additional cases per year) for 2009–2020, covering more than 95% of the population, are stored in the NDB [24,25].

The data contained in the NDB provide information on each patient's identifier (ID variable), dates of prescriptions and visits, age group, gender, region where procedures were performed, description of these procedures, World Health Organization (WHO) International Classification of Diseases (ICD-10) diagnosis codes, and medical care received. Nevertheless, this database does not include obstetric information such as laboratory data or gestational age.

We obtained approval from the MHLW to obtain medical insurance data for patients who visited medical institutions in Hokkaido between April 2010 and March 2020 (approval number: 0319). Additionally, the study was approved by the Ethics Committee of Sapporo Medical University (July 2020, Approval No. 2-1-10). The need for informed consent was waived because all data were provided to the authors in anonymized form, so the need for informed consent was waived by The Ethics Committee of Sapporo Medical University. The handling of the data complied with the guidelines [26] indicated by the MHLW. The date of access to NDB data for the purpose of this study is 13 August 2021.

### Data preparation

Two main IDs (ID1 and ID2) are used in the NDB as variables that link multiple receipts of the same patient. ID1 is based on the insurer number, insurance card number, date of birth, and gender, while ID2 is based on the name, date of birth, and gender information, each of which is encrypted and attached. In Japan, an insured person may switch insurers due to a change in the workplace or a change in family name upon marriage. This implies that there will be a certain number of individuals with the same condition but different IDs during the follow-up period. Therefore, it has been noted that one of the problems with analysis by the NDB is counting ID1 and ID2, as they exceed the actual number of patients. Attempts to more accurately count patients on the NDB have been reported, including studies that created ID0 and virtual patient identifier to count patients [27,28], a study to count the number of patients by integrating monthly bills issued by each institution for each individual when ID1 and ID2 are the same [29], and a study that counted ID1s, treating different ID1s as identical if they shared the same ID2, etc [30]. However, in all these methods, true information cannot be obtained because it is impossible to match with actual patient data, and there are limits to the pursuit of accuracy. In this study, IDT was created by concatenating ID1 and ID2 as much as possible. Even if the ID1 is different, if the ID2 is the same, it is considered to be the same patient, and even if the ID2 is different, if the ID1 is the same, it is considered to be the same patient (or the ID is reassigned so that it can be considered to be the same). In practice, the following procedure is used.

STEP 0. All data rows have the ID1, ID2, and IDT columns. Initially, IDT is empty.

STEP 1. Assign the same IDT to the same ID1.

STEP 2. Make sure that the same IDT is used for the same ID2.

STEP 3. Make sure that the same IDT is used for the same ID1.

STEP 4. Repeat steps 2 and 3 until the number of updates reaches zero.

The IDT is the theoretical lower limit of the number of persons to be collated, and the upper limit of the number of persons to be collated (the number of ID1 or ID2 counted) is also shown, thus allowing a range of accuracy to be pursued. We attempted to pursue accuracy with the IDT.

### Definition of HDP patients

In Japan, HDP is classified into four forms: gestational hypertension (GH), preeclampsia (PE), superimposed preeclampsia (SPE), and chronic hypertension (CH) [31,32]. This complies with the international definitional classification and was revised in 2018. In this study, the researchers reviewed and determined 15 Japanese standardized disease codes. These diseases do not include related diseases, such as eclampsia or HELLP syndrome, and are classified into four disease types: GH, PE, SPE, and CH. The researchers decided to treat patients with HDP if they had at least one of these 15 disease codes, and the “medical treatment start date” information was included in the receipts for the same month. The injury/disease name, injury/disease code, and corresponding International Classification of Diseases, 10th revision (ICD-10 code) are shown in Table 1 below.

**Table 1:** Number of Patients by HDP Disease Code, 2011–2019.

Disease Code	ICD-10 Description	ICD-10 Code	2011	2012	2013	2014	2015	2016	2017	2018	2019
8845462	Pre-existing essential hypertension complicating pregnancy, childbirth and the puerperium	O100	–	12	19	14	19	22	27	21	25
8845458	Pre-existing hypertensive heart disease complicating pregnancy, childbirth and the puerperium	O101	–	–	–	–	–	–	–	–	–
8845460	Pre-existing hypertensive renal disease complicating pregnancy, childbirth and the puerperium	O102	–	–	–	–	–	–	–	–	–
8845459	Pre-existing hypertensive heart and renal disease complicating pregnancy, childbirth and the puerperium	O103	–	–	–	–	–	–	–	–	–
8845461	Pre-existing secondary hypertension complicating pregnancy, childbirth and the puerperium	O104	–	–	–	–	–	–	–	–	–
8842687	Pre-eclampsia superimposed on chronic hypertension	O11	12	16	13	16	19	15	27	21	17
6429003	Gestational hypertension	O13	406	402	363	389	469	382	374	416	382
8842709	Mild to moderate pre-eclampsia	O140	54	76	104	106	104	102	128	122	131
8848335	Mild to moderate pre-eclampsia	O140	–	–	–	–	–	–	–	17	24
8842765	Severe pre-eclampsia	O141	122	150	204	200	245	267	279	257	355
8848357	Severe pre-eclampsia	O141	–	–	–	–	–	21	30	38	54
8842791	Pre-eclampsia, unspecified	O149	–	–	–	–	–	–	–	–	–
8842804	Pre-eclampsia, unspecified	O149	–	–	–	–	–	–	12	–	11
8842827	Pre-eclampsia, unspecified	O149	1935	1857	2080	1943	1943	2008	2080	2059	1682
8842828	Pre-eclampsia, unspecified	O149	143	82	103	139	207	224	219	234	246

This table shows the number of patients with HDP with each diagnosis categorized by domestic diagnosis codes for healthcare claims in Japan. The Japanese standardized disease codes are part of a comprehensive classification system used in Japan to identify and record diseases and conditions. These codes are linked to the International Classification of Diseases, 10th Revision (ICD-10), but include additional details to accommodate Japan’s healthcare system and billing practices. While some ICD-10 codes directly map to a single Japanese standardized disease code, others may correspond to multiple codes depending on the context and specific details recorded in medical receipts. For instance, codes for gestational hypertension and preeclampsia may vary based on the level of detail provided in the diagnosis or treatment description. To ensure consistency, this study reviewed and standardized 15 Japanese disease codes explicitly associated with hypertensive disorders in pregnancy (HDP). The mapping process involved careful evaluation of the disease descriptions and their correspondence to ICD-10 codes. This approach ensures that the selected codes accurately represent HDP cases within the constraints of the NDB. According to MHLW’s rules for publication of NDB data, we did not show the number of cases in categories with less than 10 (indicated by “-” in the table). The sum of patients is not equal to the total number of patients because some patients are given two or more diagnoses.

HDP; Hypertensive Disorders of Pregnancy, MHLW; the Japanese Ministry of Health, Labor and Welfare, NDB; National Database.

Next, to count HDP patients, we defined one gestational period on receipt. Because this study included data collected over 9 years, it included one woman who had multiple pregnancies and developed HDP. To count HDP due to another pregnancy as a separate patient, it is necessary to know the termination of pregnancy. However, in Japan, normal pregnancy and vaginal delivery are considered healthy life events and are not covered by insurance. Therefore, it is difficult to ascertain the termination of pregnancy from the obtained receipt information because no receipt is generated when a normal delivery occurs among those with an HDP injury or disease.

**Fig 1:**
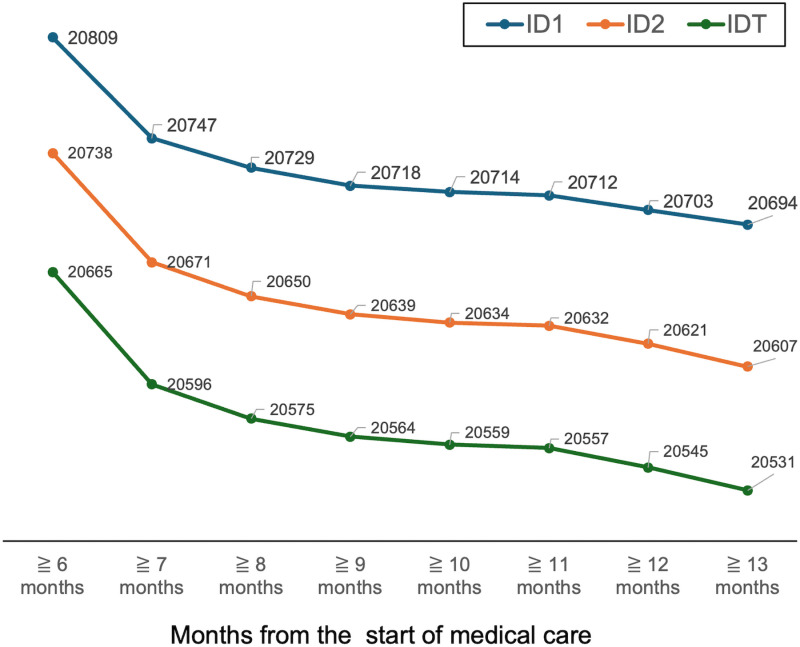
ID Counts for Periods Considered as Separate Pregnancies. ID counts were calculated and compared for separate pregnancies counted after 6, 7, 8, 9, 10, 11, 12, and 13 months from the start of medical care. When pregnancies were treated as separate starting at a 10-month interval, the difference in ID counts was the smallest between adjacent periods.

Therefore, in this study, we defined the duration of pregnancy based on the “medical care start date” information recorded on the receipts. The information on the ‘date of start of treatment’ is given not only once per pregnancy but also when the medical institution changes or the name of the injury or disease differs. Hence, there is a threshold where pregnancies with the same ID and within X months of the earliest treatment start date are counted as the same, but pregnancies over X +  1 month are counted as different pregnancies. To determine this X, the number of IDs was calculated and compared at 6, 7, 8, 9, 10, 11, 12, and 13 months after the start of treatment as separate pregnancies, and the difference in the number of IDs at approximately 10 months after counting as separate pregnancies was minimal (Fig 1). Based on the above, in this study, pregnancies within 9 months of the earliest clinical start date were defined as the same, while pregnancies of 10 months or more were considered separate pregnancies.

The number of IDs calculated by the above method was defined as the “number of HDP patients” in this study.

### Data analysis

The number of HDP patients by year and the number of patients and morbidity by age group at the start of practice from January 2011 to December 2019, before the COVID-19 epidemic, were tabulated to obtain confidence intervals and age-adjusted morbidity rates. Linear regression analysis was used for annual incidence rates, the Kruskal-Wallis test was used to test for differences in incidence rates across all age intervals, and the z-test for differences in proportions was used to analyze differences in incidence rates between specific age groups. The statistical analysis performed in this study used Python 3.12.0. The significance level was set at 5% in the statistical tests, and the Holm method correction was applied for multiple comparisons of incidence rates among age groups.

The age information provided by the NDB is based on 5-year age categories. Nevertheless, due to the MHLW’s rule that numbers less than 10 cannot be published, those aged ≤ 14 and ≥ 45, including categories with a value of less than 10, were grouped into the ≤  19 and ≥  40 age groups, respectively. Thus, the six age categories of ≤  19, 20–24, 25–29, 30–34, 35–39, and ≥  40 years were used in this study.

The incidence of HDP is determined by the number of HDP cases among pregnant women. However, since the total number of pregnant women on the NDB is unknown because the receipts do not include pregnant women with a normal pregnancy, we utilized publicly available statistical data. In Japan, the “number of pregnancy notifications” and the “number of deliveries” after 12 weeks of pregnancy are compiled, but these publicly available data do not include maternal age information. Therefore, in this study, the number of births that are close to these data and have maternal age information is used as the number of pregnant women, and the incidence rate of HDP is defined as the number of HDP cases per year as a percentage of the number of births per year. For the calculation of age-adjusted incidence rates, the average total number of births in Japan from 2011 to 2019 and the average number of births by mother's six age categories were used as the reference number of births (S1 Table). The average number was used because the change in the number of live births during this period significantly decreased, decreasing by approximately 17%.

## Results

**Fig 2:**
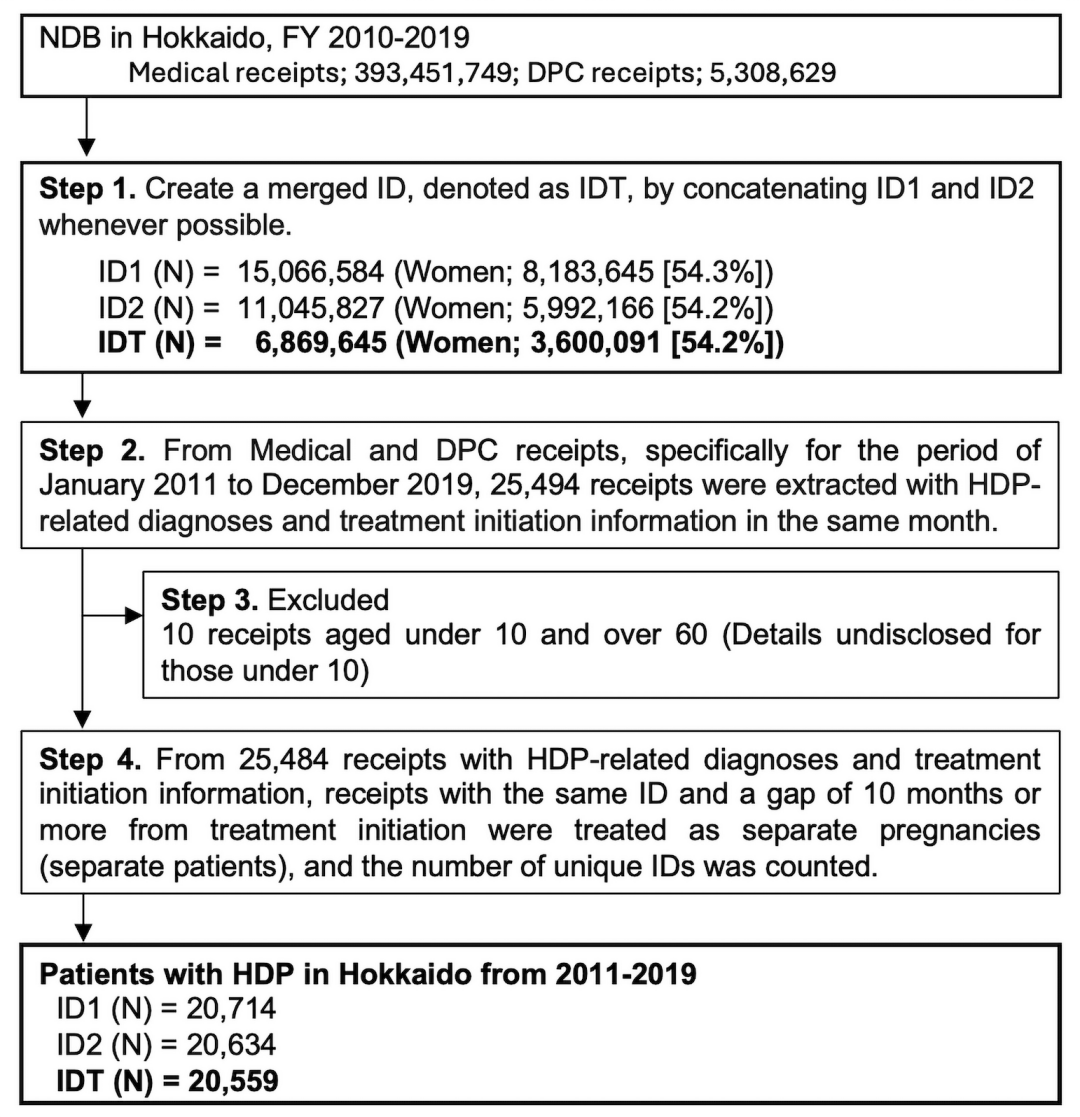
Study flowchart. In this analysis, ‘receipt serial number types’ represent the total number of receipts collected, while ‘ID types’ indicate the count of unique patient IDs. ‘Medical receipts’ include records for both outpatient and inpatient services. In contrast, ‘DPC receipts are exclusively associated with inpatient services. NDB; National Database, DPC; Diagnosis Procedure Combination, HDP; Hypertensive Disorders of Pregnancy.

The average number of HDP patients in Hokkaido in each year from 2011–2019 was determined by IDT, ID1, and ID2, with a minimum of 20,559 for IDT and a maximum of 20,714 for ID1 over the 9 years (Fig 2). The incidence rates were 6.37% for the smallest number, IDT, and 6.42% for the largest number, ID1 (Table 2). During this period, the incidence of HDP increased by 0.27% (slope =  0.003, p <  0.01), with a coefficient of determination of approximately 0.89. The number of patients in each of the 15 injury and disease categories in each year is shown in Table 1.

**Table 2:** Number of HDP Patients in Hokkaido, Japan from 2011–2019.

Number of births in Hokkaido		2011	2012	2013	2014	2015	2016	2017	2018	2019	Total
		39,292	38,686	38,190	37,058	36,696	35,129	34,058	32,642	31,020	322,771
**Number of patients with HDP** **Incidence rate** **(%)**	**IDT**	**2,156**	**2,075**	**2,268**	**2,214**	**2,395**	**2,363**	**2,446**	**2,471**	**2,171**	**20,559**
	**(%)**	**(5.49)**	**(5.36)**	**(5.94)**	**(5.97)**	**(6.53)**	**(6.73)**	**(7.18)**	**(7.57)**	**(7.00)**	**(6.37)**
	ID1	2,175	2,095	2,293	2,232	2,409	2,378	2,457	2,488	2,187	20,714
	(%)	(5.54)	(5.42)	(6.00)	(6.02)	(6.56)	(6.77)	(7.21)	(7.62)	(7.05)	(6.42)
	ID2	2,165	2,078	2,276	2,224	2,408	2,376	2,455	2,475	2,177	20,634
	(%)	(5.51)	(5.37)	(5.96)	(6.00)	(6.56)	(6.76)	(7.21)	(7.58)	(7.02)	(6.39)

The incidence rates in the table are calculated by dividing the number of patients with HDP by the total number of births in Hokkaido for each year.

HDP; Hypertensive Disorders of Pregnancy.

**Fig 3:**
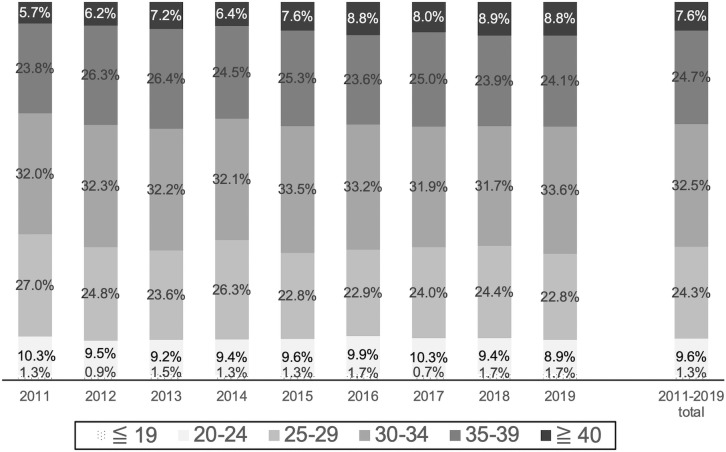
Age composition of HDP patients, 2011–2019. The chart shows the age distribution of HDP patients in Hokkaido from 2011 to 2019, with individual years and the average for the entire period. HDP; Hypertensive Disorders of Pregnancy.

**Fig 4:**
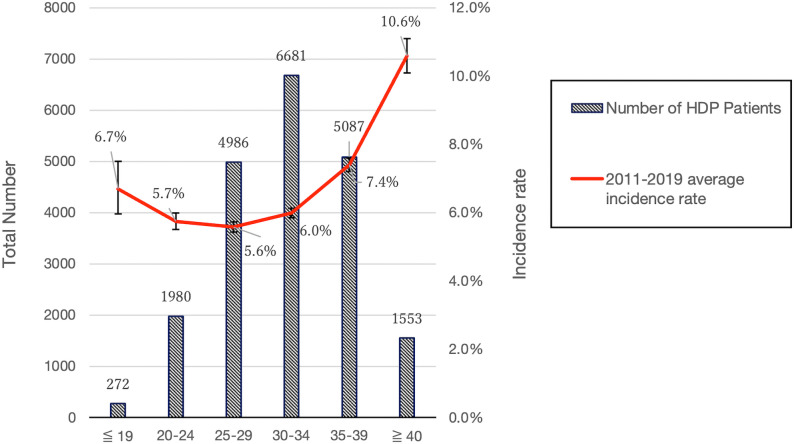
Number of HDP Patients and Average Incidence Rate. This figure presents the number of HDP patients alongside the average incidence rates for 2011–2019, with 95% confidence intervals calculated using the Wilson score method for age-specific rates. HDP; Hypertensive Disorders of Pregnancy.

The age composition of the nine-year-old HDP patients was determined by age groups ≤  19, 20–24, 25–29, 30–34, 35–39, and ≥  40 years. The results showed that the percentage of the ≥  40 age group increased from 5.7% in 2011 to 8.8% in 2019, and the percentage of the 25–29 age group decreased from 27.0% to 22.8%, while there were no significant changes in the other groups. (Fig 3). The mean incidence rates and confidence intervals for each age group over this approximate 10-year period are shown in Fig 4. The incidence rate was lowest in the 25–29 age group at 5.58% (95% confidence interval [CI]: 5.43–5.73) and increased with age, with a rate of 10.58% (95% Cl: 10.10–11.09) in the 40 + age group. On the other hand, the rate was 6.70% (95% CI: 5.97–7.51) in the group aged 19 years or younger, which was higher than that in the group aged 20 years or older. To evaluate differences in incidence rates across all age groups, a Kruskal-Wallis test was performed, and significant differences were found (Kruskal-Wallis test quantity 59.0, p <  0.01). In addition, z-tests for differences in proportions were performed between the 25–29 age group and each of the other five age groups, and multiple comparison correction was applied using the Holm method. The results showed that there was a significant difference in proportions between ≤  19 years (z =  −3.016, p =  0.0025, adjusted p =  0.0051), 20–24 years (z =  −1.118, p =  0.26, adjusted p =  0.26), 30–34 years (z =  −3.893, p <  0.0001, adjusted p =  0.00030), 35–39 years (z =  −14.715, p <  0.0001, adjusted p <  0.0001), and ≥  40 years (z =  −23.139, p <  0.0001, adjusted p <  0.0001), and there were statistically significant differences between the ≤  19, 30–34, 35–39, and ≥  40 age groups and the 25–29 age group. The data on the number of patients and incidence rates by age group for each year are presented in Table 3.

**Table 3:** Number of Patients with HDP by Age Group, 2011–2019.

Age groups (%)	2011	2012	2013	2014	2015	2016	2017	2018	2019	2011–2019 total
≦ 19	27	18	34	28	30	39	18	41	37	272
	(5.11%)	(3.39%)	(6.51%)	(5.53%)	(6.61%)	(8.65%)	(5.16%)	(10.70%)	(10.95%)	(6.70%)
20–24	221	198	208	209	230	235	253	233	193	1,980
	(4.66%)	(4.66%)	(5.02%)	(5.42%)	(6.08%)	(6.43%)	(7.23%)	(6.87%)	(6.12%)	(5.74%)
25–29	583	514	535	582	546	540	588	603	495	4,986
	(5.02%)	(4.56%)	(4.86%)	(5.68%)	(5.51%)	(5.72%)	(6.51%)	(7.01%)	(6.03%)	(5.58%)
30–34	689	671	730	711	802	784	781	783	730	6,681
	(5.15%)	(5.08%)	(5.66%)	(5.55%)	(6.26%)	(6.40%)	(6.49%)	(6.90%)	(6.76%)	(5.99%)
35–39	513	545	598	543	605	557	611	591	524	5,087
	(6.60%)	(6.81%)	(7.45%)	(6.86%)	(7.52%)	(7.41%)	(8.29%)	(8.24%)	(7.63%)	(7.41%)
≧ 40	123	129	163	141	182	208	195	220	192	1,553
	(9.82%)	(8.96%)	(10.16%)	(8.17%)	(10.71%)	(11.49%)	(11.06%)	(12.67%)	(11.69%)	(10.58%)

The percentages in the table represent the incidence rates for each age group of mothers, calculated as the number of HDP patients divided by the number of births within the same age category.

HDP; Hypertensive Disorders of Pregnancy.

**Fig 5:**
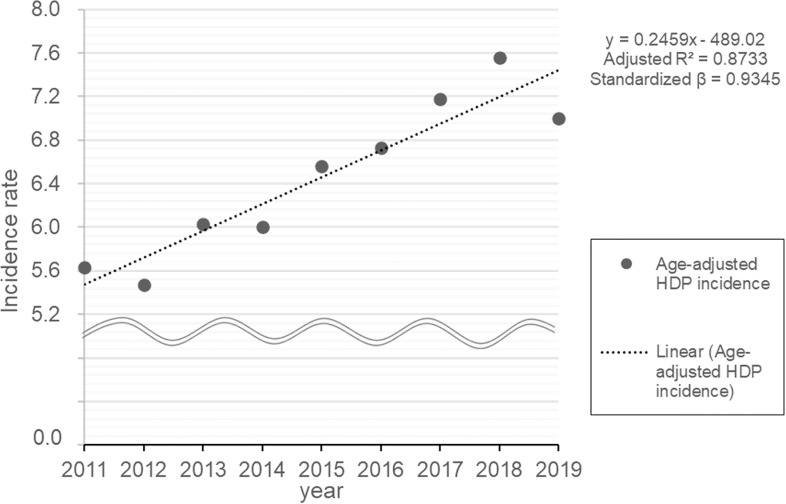
Age-adjusted HDP incidence rate. The age-adjusted incidence rates were calculated using the average birth data for six age groups ( ≤ 19, 20–24, 25–29, 30–34, 35–39, ≥  40) in Japan from 2011 to 2019. HDP; Hypertensive Disorders of Pregnancy.

The age-adjusted HDP incidence rates for this period were calculated, and the linear regression analysis results are shown in Fig 5. The age-adjusted incidence rates were 5.58% in 2011 and 6.94% in 2019, indicating an increase of 0.25% per year.

## Discussion

Using data from the National Database, which contains insurance practice data for most of the Japanese population, we were able to show age-specific patient numbers and incidence rates for HDP in the Hokkaido region of Japan from 2011–2019. To our knowledge, this is the first report of a comprehensive HDP study using this scale. Hokkaido is the largest island in Japan, accounting for 22% of the total land area, and is surrounded by the sea, making overland travel possible only by rail. Therefore, except for relocation and homecoming deliveries (the Japanese custom of returning to a woman's parents’ home during late pregnancy or childbirth to receive support from family members), there is almost no outflow of people into and out of the prefecture to receive medical care. Therefore, the annual trends in the number of HDP patients in this region over a given period may approximate the trends in the number of HDP patients in Japan, a developed country.

In this study, the number of HDP cases initiated during a year divided by the number of births during the year was treated as the HDP incidence rate, and the resulting 9-year average incidence rate was 6.37% (Table 2). According to a database created by the Japan Society of Obstetrics and Gynecology, which registers approximately 20% of births in the population of higher tertiary hospitals, there were 108,562 cases of HDP over the same 9-year period, representing 5.79% of all births after 22 weeks and 5.82% of live births [33]. The prevalence of HDP in the Hokkaido Birth Cohort Study reported by Poudel K *et al*. was 1.7%, which was lower than that in this study (6.8%) [34]. The previous study had a different disease background than the present study, with a frequency of 20% in mothers over 35 years of age, which was consistent with results based on a much older database from 2002 to 2013 [35]. Considering the retrospective trend of a gradual increase in HDP from year to year, as shown in Fig 5, as well as the margin of error due to differences in sample size, these differences are considered acceptable. For other countries, the prevalence of HDP in Ireland in 2016 was 5.9%, and a cohort study of births in the United States from 1976–1982 reported an HDP incidence of 7.3% [33,35,36]. Many reports have also fluctuated in the approximate range of 5–10%, with a review by Umesawa *et al*. reporting a rate of approximately 5.2–8.2% [14,15]. Although the diagnostic criteria and classifications of HDP have varied slightly over time and from country to country, the methods used to calculate incidence rates in various studies have not been completely consistent. Nevertheless, even considering this, the results of this study are within the range of those of previous reports and support the validity of the present data.

The findings of this study highlight significant age differences in the incidence of HDP in Hokkaido. In this study, the mean incidence rate by age group was 5.58% (95% CI: 5.43–5.73), with the lowest in the 25–29 age group, 10.58% (95% CI: 10.10–11.09) in the over 40 age group, and even 6.70% (95% CI: 5.97–7.51) in the under 19 age group, which was significantly greater than that of the 25–29 age group (Fig 4). A study analyzing HDP incidence rates in 204 countries around the world reported that the lowest estimated incidence rate was in the 25–29 age group, with higher rates in younger and older age groups [16], and similar results were obtained in this highly universal study limited to Japan, a developed country. The increased risk of HDP in women over 40 years of age, and even in women under 20 years of age, suggests the existence of a multifaceted risk profile that goes beyond simple demographic categories. This “J-curve” phenomenon in morbidity contradicts conventional risk perceptions and indicates that factors other than maternal aging may contribute to the development of HDP. It has already been reported that maternal advanced age is a risk factor for hypertension during pregnancy [15,17–19,37], and study reports on teenage morbidity, including those from developing countries with high rates of young childbearing, have reported a greater risk of HDP and eclampsia compared to those in their 20s [16,38,39]. On the other hand, there are few reports on HDP in teenagers, especially in developed countries. In Japan, young pregnancies account for less than 1% of all pregnancies, but there are many sociologically high-risk pregnancies [40,41], especially smoking, which is known to increase the risk of developing HDP [42,43]; however, the smoking rate among pregnant teenage women is high [44,45], and Hokkaido is the region with the highest female smoking rate in Japan [46]. Another reason for the higher incidence among young women may be the high smoking rate and inadequate education for young pregnant women. The pathogenesis of HDP is known to involve immune tolerance deficiency [47], uterine spiral artery remodeling defects [48], and decreased blood flow and hypoxia in the placental region due to increased production of the anti-angiogenic factors soluble fms-like tyrosine kinase 1 (sFlt-1) [48] and soluble endoglin (sEng) [49–51]. Indeed, significant angiogenic factor imbalances have been reported in pregnant women who develop HDP. While previous reports indicate that the production of sFlt-1, a protein associated with various health conditions, is affected by factors such as advanced glycation end products (AGEs) in elderly individuals [52], the relationship between young pregnancy and sFlt-1 remains unclear. Taken together, it can be inferred that while new factors that may explain the J-curve phenomenon need to be scrutinized, risky health behaviors are related in no small way to the development of HDP and should receive special attention.

According to the current analysis of NDB data for the 9 years before the COVID-19 epidemic, the incidence of HDP increased even after age adjustment (Fig 5). Although the incidence increased to 8.8% in 2019, the increase in incidence even after age adjustment suggests that the main reason is a factor other than gestational age. A retrospective cohort study using the U.S. birth database reported that the prevalence of HDP increased from 4.5% in 2014 to 6.0% in 2018, a trend similar to our results [53]. A study examining the prevalence of HDP in urban and rural areas of the United States also reported a doubling of prevalence in both regions between 2007 and 2019, the study period [54]. One of the factors contributing to the annual increase in incidence in Japan is the increase in assisted reproductive technology (ART), which has been reported to have a significantly greater incidence of HDP than spontaneous conception [55,56]. In 2011, ART births accounted for 3.1% of all births in Japan, but this percentage has increased to 7.0% over the past decades [57]. It should be emphasized that in the future, we can expect to see an increasing number of pregnancies on ART and an increasing number of HDP patients in developed countries.

In recent years, appropriate management of HDP has reduced severe effects, such as maternal mortality. Nevertheless, it has been reported that a 10 mmHg increase in mean diastolic blood pressure during pregnancy increases the risk of hypertension later in life by 1.7-fold, making HDP a subject that needs to be followed throughout pregnancy and later in life, regardless of its severity [58]. It is necessary to pay attention to the increasing trend of HDP incidence in Japan and worldwide and to link this to public health measures in each country, especially in terms of maternal and child health.

### Strengths and limitations

This study addresses a significant gap in existing research by evaluating the incidence of HDP among younger populations, a group that has been largely overlooked in previous studies. Existing research has predominantly focused on older pregnant women or general population-based estimates, often lacking detailed age-stratified analyses. For instance, international studies such as the Global Burden of Disease project have provided valuable insights into overall prevalence trends but have not adequately captured rare conditions like HDP in adolescents. In contrast, NDB allows for comprehensive analyses across a broad range of age groups, including younger individuals, enabling the detailed identification of rare cases and age-specific trends. Leveraging this capability, the present study tracked HDP incidence rates in younger populations over nine years, addressing a critical gap in public health research.

Moreover, one of the strengths of the NDB lies in its foundation on Japan’s universal health insurance system, which covers nearly the entire population. This minimizes selection bias while ensuring robust statistical power. The NDB has been widely recognized as a valuable resource for understanding healthcare utilization trends and disease incidence [28–30,59,60] By utilizing real-world clinical data, it provides critical insights into disease management and patient behavior, complementing findings from prospective cohort studies. This study harnessed these features of the NDB to calculate HDP incidence rates in the Hokkaido region, offering a unique perspective on the epidemiology of HDP.

Another notable strength of this study is its ability to accurately track the annual trends of HDP over a long period, from 2011 to 2019, prior to the onset of the COVID-19 pandemic. Importantly, the study also includes data covering pregnancies among women aged 19 years or younger, an age group that has been challenging to analyze in prior prospective cohort studies conducted in developed countries. This comprehensive inclusion of younger age groups highlights the distinct contribution of this study to understanding the epidemiology of HDP across diverse demographic categories.

However, our study has several limitations. First, the study did not include specific tests or prescriptions and treated patients with HDP, such as those who had any of the 15 injury or disease names and medical care initiation information in the same month. Therefore, it was impossible to classify severity or disease type, and the number of patients included in the study ranged from mild patients who did not require treatment to severe patients. Second, the NDB data used in this study did not include detailed data such as patient symptoms, blood pressure readings, or laboratory results. Therefore, patients with HDP-related injuries and illnesses were considered HDP patients. Nevertheless, it was not possible to confirm whether the injury or illness name correctly reflected the diagnosis, and no validation study was conducted. Third, since normal delivery was not covered by insurance, the date of delivery could not be determined from the receipt information, and the duration of pregnancy was uniformly defined as the same pregnancy from the start date of medical care to nine months. This is illustrated in Fig 1 for clarity, but the number of HDP patients may differ depending on the setting of this cutoff value. Fourth, the lack of data on patient background makes it difficult to estimate factors associated with increased morbidity. Despite these limitations, this study is significant because it is the world's first long-term all-inclusive survey of a specific geographic region, and it allowed us to determine the incidence rates by age over nine years. Finally, the data used in this study are electronic data obtained from insured patients and do not include data on welfare recipients who do not use insurance or on receipts that are not electronic. Since the percentage of welfare recipients [61] and the rate of electronic receipt [24,62] did not change significantly between 2011 and 2019, we believe that the impact of these changes can be treated as negligible. In addition, the definitional classification of HDP changed in Japan in 2018; however, we infer that this effect is also not significant because we treated all the data based on that classification.

## Conclusion

Analysis using the Japanese Health Insurance Database revealed that the average incidence of HDP in 2011 and 2019 was 6.37%, with the lowest incidence in the 25–29 age group and a “J- curve” with a greater incidence in the younger and older age groups. Age-adjusted morbidity increased slowly over this period, suggesting an association with factors other than older maternal age. Further approaches to identify factors, as well as interventions for existing risk factors, are needed for this disease, which is expected to be on the rise worldwide.

## Supporting information

S1 Table Number of Pregnancy Notifications, Deliveries, and Age-specific Births in Hokkaido. This table presents the number of pregnancy notifications, delivery cases, births, and age-specific birth counts of mothers in Hokkaido, based on publicly available data from 2011 to 2019. It includes annual figures for each year as well as the average over the period. The rightmost column displays the national averages for Japan from 2011 to 2019.
(TIFF)

## References

[1] Say L, Chou D, Gemmill A, Tuncalp O, Moller AB, Daniels J, et al. Global causes of maternal death: a WHO systematic analysis. Lancet Glob Health. 2014;2(6):e323–333. doi: 10.1016/S2214-109X(14)70227-X 25103301

[2] Wu P, Green M, Myers JE. Hypertensive disorders of pregnancy. BMJ. 2023;381:e071653. doi: 10.1136/bmj-2022-071653 37391211

[3] Metoki H, Iwama N, Hamada H, Satoh M, Murakami T, Ishikuro M, et al. Hypertensive disorders of pregnancy: definition, management, and out-of-office blood pressure measurement. Hypertens Res. 2022;45(8):1298–309. doi: 10.1038/s41440-022-00965-6 35726086 PMC9207424

[4] Giorgione V, Ridder A, Kalafat E, Khalil A, Thilaganathan B. Incidence of postpartum hypertension within 2 years of a pregnancy complicated by pre-eclampsia: a systematic review and meta-analysis. BJOG. 2021;128(3):495–503. doi: 10.1111/1471-0528.16545 32981216

[5] Behrens I, Basit S, Melbye M, Lykke JA, Wohlfahrt J, Bundgaard H, et al. Risk of post-pregnancy hypertension in women with a history of hypertensive disorders of pregnancy: nationwide cohort study. BMJ. 2017;358:j3078–j3078. doi: 10.1136/bmj.j3078 28701333 PMC5506851

[6] Watanabe M, Sairenchi T, Nishida K, Uchiyama K, Haruyama Y, Satonaka H, et al. Gestational hypertension as risk factor of hypertension in middle-aged and older women. Int J Environ Res Public Health. 2020;17(11):4052. doi: 10.3390/ijerph17114052 32517151 PMC7312590

[7] Wagata M, Kogure M, Nakaya N, Tsuchiya N, Nakamura T, Hirata T, et al. Hypertensive disorders of pregnancy, obesity, and hypertension in later life by age group: a cross-sectional analysis. Hypertens Res. 2020;43(11):1277–83. doi: 10.1038/s41440-020-0463-8 32404963

[8] Sukmanee J, Liabsuetrakul T. Risk of future cardiovascular diseases in different years postpartum after hypertensive disorders of pregnancy: a systematic review and meta-analysis. Medicine (Baltim). 2022;101(30):e29646. doi: 10.1097/MD.0000000000029646 35905265 PMC9333537

[9] Riise HKR, Sulo G, Tell GS, Igland J, Nygård O, Iversen AC, et al. Association between gestational hypertension and risk of cardiovascular disease among 617,589 Norwegian women. J Am Heart Assoc. 2018;7(10):e008337. doi: 10.1161/JAHA.117.008337 29755034 PMC6015305

[10] Tooher J, Thornton C, Makris A, Ogle R, Korda A, Hennessy A. All hypertensive disorders of pregnancy increase the risk of future cardiovascular disease. Hypertension. 2017;70(4):798–803. doi: 10.1161/HYPERTENSIONAHA.117.09246 28893895

[11] Stuart JJ, Tanz LJ, Rimm EB, Spiegelman D, Missmer SA, Mukamal KJ, et al. Cardiovascular risk factors mediate the long-term maternal risk associated with hypertensive disorders of pregnancy. J Am Coll Cardiol. 2022;79(19):1901–13. doi: 10.1016/j.jacc.2022.03.335 35550687 PMC9176211

[12] Grandi SM, Filion KB, Yoon S, Ayele HT, Doyle CM, Hutcheon JA, et al. Cardiovascular disease-related morbidity and mortality in women with a history of pregnancy complications. Circulation. 2019;139(8):1069–79. doi: 10.1161/CIRCULATIONAHA.118.036748 30779636

[13] Watanabe K, Kimura C, Iwasaki A, Mori T, Matsushita H, Shinohara K, et al. Pregnancy-induced hypertension is associated with an increase in the prevalence of cardiovascular disease risk factors in Japanese women. Menopause. 2015;22(6):656–9. doi: 10.1097/GME.0000000000000361 25387344

[14] Hutcheon JA, Lisonkova S, Joseph KS. Epidemiology of pre-eclampsia and the other hypertensive disorders of pregnancy. Best Pract Res Clin Obstet Gynaecol. 2011;25(4):391–403. doi: 10.1016/j.bpobgyn.2011.01.006 21333604

[15] Umesawa M, Kobashi G. Epidemiology of hypertensive disorders in pregnancy: prevalence, risk factors, predictors and prognosis. Hypertens Res. 2017;40(3):213–20. doi: 10.1038/hr.2016.126 27682655

[16] Wang W, Xie X, Yuan T, Wang Y, Zhao F, Zhou Z, et al. Epidemiological trends of maternal hypertensive disorders of pregnancy at the global, regional, and national levels: a population‐based study. BMC Pregnancy Childbirth. 2021;21(1): doi: 10.1186/s12884-021-03809-2 PMC8106862 33964896

[17] Londero AP, Rossetti E, Pittini C, Cagnacci A, Driul L. Maternal age and the risk of adverse pregnancy outcomes: a retrospective cohort study. BMC Pregnancy Childbirth. 2019;19(1):261. doi: 10.1186/s12884-019-2400-x 31337350 PMC6651936

[18] Ye C, Ruan Y, Zou L, Li G, Li C, Chen Y, et al. The 2011 survey on hypertensive disorders of pregnancy (HDP) in China: prevalence, risk factors, complications, pregnancy and perinatal outcomes. PLoS One. 2014;9(6):e100180. doi: 10.1371/journal.pone.0100180 24937406 PMC4061123

[19] Ogawa K, Urayama KY, Tanigaki S, Sago H, Sato S, Saito S, et al. Association between very advanced maternal age and adverse pregnancy outcomes: a cross sectional Japanese study. BMC Pregnancy Childbirth. 2017;17(1). doi: 10.1186/s12884-017-1540-0 PMC5635576 29017467

[20] Statistics Bureau of Japan. Vital statistics survey: final counts of births [Data file]. (in Japanese). 2021 [cited 2023 Nov 20]. Available from: https://www.e-stat.go.jp/dbview?sid=0003411610

[21] Fulmer S. World population review, 2024 [cited 2024 Mar 19]. Available from: https://worldpopulationreview.com/country-rankings/average-age-of-having-first-child-by-country

[22] Matsuda S. Health policy in Japan – current situation and future challenges. JMA J. 2019;2(1):1–10. doi: 10.31662/jmaj.2018-0016 33681508 PMC7930804

[23] Japan Ministry of Health Labour, and Welfare. Survey on welfare recipients (in Japanese). Available from: https://www.mhlw.go.jp/toukei/list/74-16.html Accessed 20 Nov 2023

[24] Japan Ministry of Health Labour, and Welfare. Website for the Japan National database of health insurance claims. (in Japanese). [cited 2024 Mar 11]. Available from: https://www.mhlw.go.jp/stf/seisakunitsuite/bunya/kenkou_iryou/iryouhoken/reseputo/index.html

[25] Tsuneishi M, Yamamoto T, Yamaguchi T, Kodama T, Sato T. Association between number of teeth and Alzheimer’s disease using the National Database of Health Insurance Claims and Specific Health Checkups of Japan. PLoS One. 2021;16(4):e0251056. doi: 10.1371/journal.pone.0251056 33930067 PMC8087029

[26] Guidelines for the use of the national database of health insurance claims and specific health checkups of Japan, Second Edition. (in Japanese). [cited 2023 Nov 20]. Available from: https://www.mhlw.go.jp/content/12400000/001158704.pdf

[27] Kubo S, Noda T, Myojin T, Nishioka Y, Higashino T, Matsui H, et al. National database of health insurance claims and specific health checkups of Japan (NDB): outline and patient-matching technique. BioRxiv. 2018;1:280008. doi: 10.1101/280008

[28] Kido A, Miyake M, Tamura H, Hiragi S, Kimura T, Ohtera S, et al. Incidence of central serous chorioretinopathy (2011–2018): a nationwide population-based cohort study of Japan. Br J Ophthalmol. 2022;106(12):1748–53. doi: 10.1136/bjophthalmol-2021-319403 34261662 PMC9685711

[29] Maeda E, Ishihara O, Tomio J, Miura H, Kobayashi Y, Terada Y, et al. Cesarean delivery rates for overall and multiple pregnancies in Japan: a descriptive study using nationwide health insurance claims data. J Obstet Gynaecol Res. 2021;47(6):2099–109. doi: 10.1111/jog.14772 33779012

[30] Den H, Ito J, Kokaze A. Epidemiology of developmental dysplasia of the hip: analysis of Japanese national database. J Epidemiol. 2021;33(4):186–92. doi: 10.2188/jea.je20210074 PMC9939923 34380918

[31] Watanabe K, Matsubara K, Nakamoto O, Ushijima J, Ohkuchi A, Koide K, et al. Outline of the new definition and classification of “Hypertensive Disorders of Pregnancy (HDP)”; a revised JSSHP statement of 2005. Hypertens Res Pregnancy. 2018;6(2):33–7. doi: 10.14390/jsshp.hrp2018-014

[32] Makino S, Takeda J, Takeda S, Watanabe K, Matsubara K, Nakamoto O, et al. New definition and classification of “Hypertensive Disorders of Pregnancy (HDP). Hypertens Res Pregnancy. 2019;7(1):1–5. doi: 10.14390/jsshp.hrp2019-010

[33] The Japan Society of Obstetrics and Gynecology. Report of the Perinatal Committee. (in Japanese). [cited 2024 Mar 7]. Available from: https://www.jsog.or.jp/medical/627/

[34] Poudel K, Kobayashi S, Miyashita C, Ikeda-Araki A, Tamura N, Ait Bamai Y, et al. Hypertensive Disorders during Pregnancy (HDP), maternal characteristics, and birth outcomes among Japanese women: a Hokkaido study. Int J Environ Res Public Health. 2021;18(7):3342. doi: 10.3390/ijerph18073342 33804885 PMC8038052

[35] Garovic VD, White WM, Vaughan L, Saiki M, Parashuram S, Garcia-Valencia O, et al. Incidence and long-term outcomes of hypertensive disorders of pregnancy. J Am Coll Cardiol. 2020;75(18):2323–34. doi: 10.1016/j.jacc.2020.03.028 32381164 PMC7213062

[36] Corrigan L, O’Farrell A, Moran P, Daly D. Hypertension in pregnancy: prevalence, risk factors and outcomes for women birthing in Ireland. Pregnancy Hypertens. 2021;24:1–6. doi: 10.1016/j.preghy.2021.02.005 33618054

[37] Cavazos-Rehg PA, Krauss MJ, Spitznagel EL, Bommarito K, Madden T, Olsen MA, et al. Maternal age and risk of labor and delivery complications. Matern Child Health J. 2015;19(6):1202–11. doi: 10.1007/s10995-014-1624-7 25366100 PMC4418963

[38] Ganchimeg T, Ota E, Morisaki N, Laopaiboon M, Lumbiganon P, Zhang J, et al; WHO Multicountry Survey on Maternal Newborn Health Research Network. Pregnancy and childbirth outcomes among adolescent mothers: a World Health Organization multicountry study. BJOG. 2014;121(Suppl 1):40–8. doi: 10.1111/1471-0528.12630 24641534

[39] Abalos E, Cuesta C, Carroli G, Qureshi Z, Widmer M, Vogel JP, et al; WHO Multicountry Survey on Maternal and Newborn Health Research Network. Pre-eclampsia, eclampsia and adverse maternal and perinatal outcomes: a secondary analysis of the World Health Organization Multicountry Survey on Maternal and Newborn Health. BJOG. 2014;121(Suppl 1):14–24. doi: 10.1111/1471-0528.12629 24641531

[40] Statistics Bureau of Japan. Number, percentage, and fertility rate of live births by mother’s age (5-year age group) by year [Data file]. (in Japanese) Accessed 18 Mar 2024.

[41] Akazawa M, Hashimoto K. The analysis of social background and perinatal prognosis of teen pregnancy in Japan (in Japanese). J Jpn Soc Perinat Neonatal Med. 2023;59(2):194–9. doi: 10.34456/jjspnm.59.2_194

[42] Tanaka K, Nishigori H, Watanabe Z, Iwama N, Satoh M, Murakami T, et al; Japan Environment & Children’s Study Group. Higher prevalence of hypertensive disorders of pregnancy in women who smoke: the Japan environment and children’s study. Hypertens Res. 2019;42(4):558–66. doi: 10.1038/s41440-019-0206-x 30662062

[43] Morisaki N, Obara T, Piedvache A, Kobayashi S, Miyashita C, Nishimura T, et al. Association between smoking and hypertension in pregnancy among Japanese Women: a meta-analysis of birth cohort studies in the Japan Birth Cohort Consortium (JBiCC) and JECS. J Epidemiol. 2023;33(10):498–507. doi: 10.2188/jea.JE20220076 35934789 PMC10483100

[44] Suzuki S. Clinical significance of pregnancy in adolescence in Japan. J Matern Fetal Neonatal Med. 2019;32(11):1864–8. doi: 10.1080/14767058.2017.1421928 29278958

[45] Ishitsuka K, Yamamoto-Hanada K, Ayabe T, Mezawa H, Konishi M, Saito-Abe M, et al; Japan Environment and Children's Study Group. Risky health behaviors of teenage mothers and infant outcomes in the Japan environment and children’s study: a nationwide cohort study. J Pediatr Adolesc Gynecol. 2019;32(2):146–52. doi: 10.1016/j.jpag.2018.10.009 30395983

[46] Hokkaido Health and Welfare Department. Smoking situation in Hokkaido. (in Japanese). [cited 2024 Mar 19]. Available from: https://www.pref.hokkaido.lg.jp/hf/kth/kak/tkh/framepage/kituennjyoukyou.html

[47] Rana S, Lemoine E, Granger JP, Karumanchi SA. Preeclampsia. Circ Res. 2019;124(7):1094–112. doi: 10.1161/CIRCRESAHA.118.313276 30920918

[48] Sircar M, Thadhani R, Karumanchi SA. Pathogenesis of preeclampsia. Curr Opin Nephrol Hypertens. 2015;24(2):131–8. doi: 10.1097/MNH.0000000000000105 25636145

[49] El-Sayed AAF. Preeclampsia: a review of the pathogenesis and possible management strategies based on its pathophysiological derangements. Taiwan J Obstet Gynecol. 2017;56(5):593–8. doi: 10.1016/j.tjog.2017.08.004 29037542

[50] Pankiewicz K, Szczerba E, Maciejewski T, Fijałkowska A. Non-obstetric complications in preeclampsia. Prz Menopauzalny. 2019;18(2):99–109. doi: 10.5114/pm.2019.85785 31485207 PMC6719635

[51] Phipps E, Prasanna D, Brima W, Jim B. Preeclampsia: updates in pathogenesis, definitions, and guidelines. Clin J Am Soc Nephrol. 2016;11(6):1102–13. doi: 10.2215/CJN.12081115 27094609 PMC4891761

[52] Huang QT, Zhang M, Zhong M, Yu YH, Liang WZ, Hang LL, et al. Advanced glycation end products as an upstream molecule triggers ROS-induced sFlt-1 production in extravillous trophoblasts: a novel bridge between oxidative stress and preeclampsia. Placenta. 2013;34(12):1177–82. doi: 10.1016/j.placenta.2013.09.017 24144948

[53] Fronek E, Martins S, Contag S. Prevalence of hypertensive disorders of pregnancy at or beyond 39 weeks gestational age and associated maternal complications. Hypertens Pregnancy. 2023;42(1):2217452. doi: 10.1080/10641955.2023.2217452 37272659

[54] Cameron NA, Everitt I, Seegmiller LE, Yee LM, Grobman WA, Khan SS. Trends in the incidence of new‐onset hypertensive disorders of pregnancy among rural and urban areas in the United States, 2007 to 2019. J Am Heart Assoc. 2022;11(2):e023791. doi: 10.1161/JAHA.121.023791 35014858 PMC9238536

[55] Qin J, Liu X, Sheng X, Wang H, Gao S. Assisted reproductive technology and the risk of pregnancy-related complications and adverse pregnancy outcomes in singleton pregnancies: a meta-analysis of cohort studies. Fertil Steril. 2016;105(1):73–85.e1. doi: 10.1016/j.fertnstert.2015.09.007 26453266

[56] Omani-Samani R, Alizadeh A, Almasi-Hashiani A, Mohammadi M, Maroufizadeh S, Navid B, et al. Risk of preeclampsia following assisted reproductive technology: systematic review and meta-analysis of 72 cohort studies. J Matern Fetal Neonatal Med. 2020;33(16):2826–40. doi: 10.1080/14767058.2018.1560406 30563382

[57] The Japan Society of Obstetrics and Gynecology. ART Data Book (in Japanese). [cited 2024 Mar 12]. Available from: https://www.jsog.or.jp/medical/641/

[58] Iino K, Higuchi T, Ogawa M, Yamauchi Y, Misaki N, Tanaka K, et al. Blood pressure during pregnancy is a useful predictive maker for hypertension and dyslipidemia later in life, a population-based, cross-sectional study. Maturitas. 2016;87:84–8. doi: 10.1016/j.maturitas.2016.02.012 27013293

[59] Suto M, Sugiyama T, Imai K, Furuno T, Hosozawa M, Ichinose Y, et al. Studies of health insurance claims data in Japan: a scoping review. JMA J. 2023;6(3):233–45. doi: 10.31662/jmaj.2022-0184 37560376 PMC10407298

[60] Hirose N, Ishimaru M, Morita K, Yasunaga H. A review of studies using the Japanese national database of health insurance claims and specific health checkups. Ann Clin Epidemiol. 2020;2(1):13–26. doi: 10.37737/ace.2.1_13

[61] Hokkaido Health and Welfare Department, Welfare Bureau, Community Welfare Division, Summary of welfare assistance implementation (Fiscal Year 2021 Results)(in Japanese). [cited 2023 Dec 11]. Available from: https://www.pref.hokkaido.lg.jp/hf/feg/hog/hogoanai4.html

[62] Social Insurance Medical Payment Fund. Status of claims by type of claim form. (in Japanese). [cited 2023 Nov 20]. Available from: https://www.ssk.or.jp/smph/tokeijoho/tokeijoho_rezept/

