# Supplementary figures and images for: Maternal age and the rising incidence of hypertensive disorders of pregnancy: A comprehensive analysis of national claims data from Japan
Source: PLoS One. 2025 Feb 20;20(2):e0319177. doi: 10.1371/journal.pone.0319177 (PMC11841901; doi:10.1371/journal.pone.0319177)

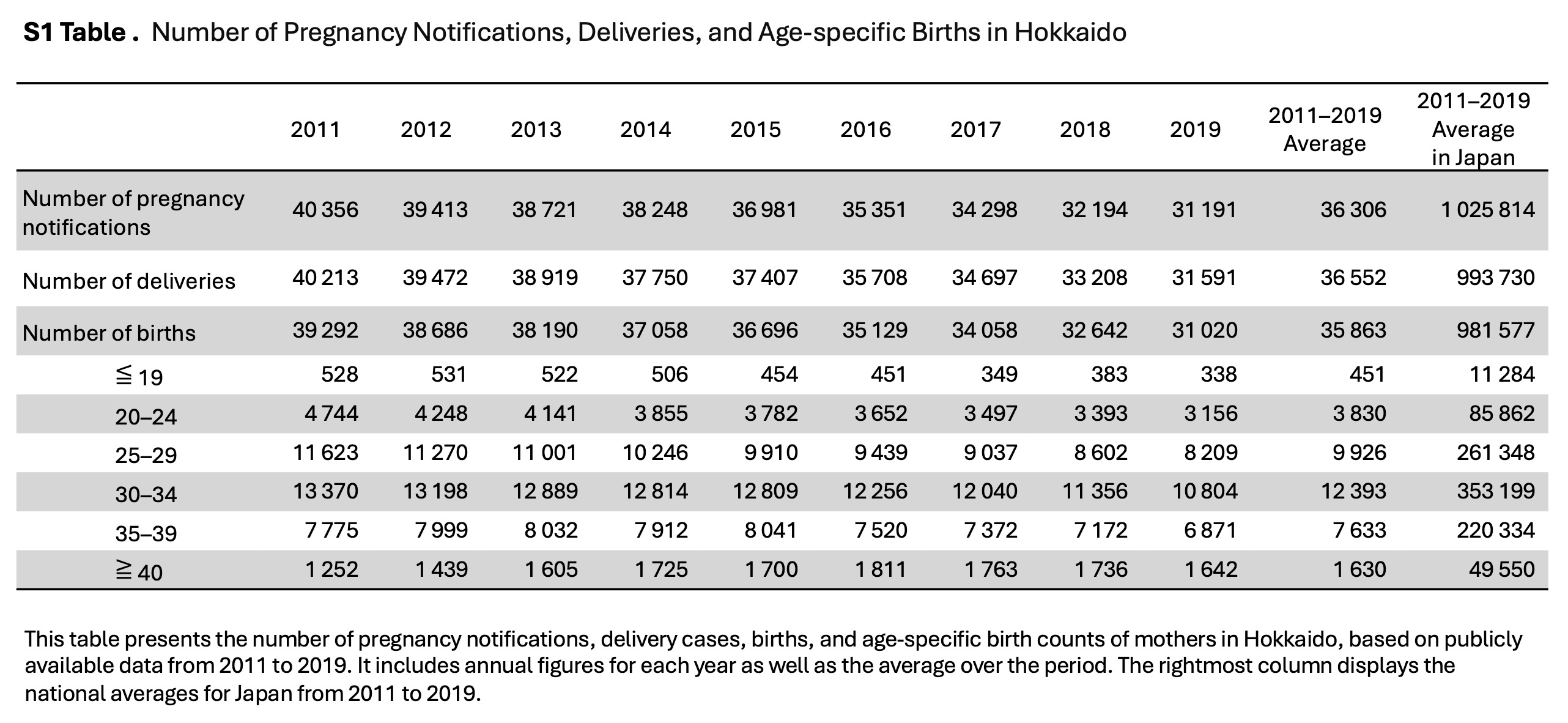

Supplement: S1 Table — This table presents the number of pregnancy notifications, delivery cases, births, and age-specific birth counts of mothers in Hokkaido, based on publicly available data from 2011 to 2019. It includes annual figures for each year as well as the average over the period. The rightmost column displays the national averages for Japan from 2011 to 2019. (TIFF) [file pone.0319177.s001.tiff]
