# Supplementary material for: Maternal age and the rising incidence of hypertensive disorders of pregnancy: A comprehensive analysis of national claims data from Japan
Source: PLoS One. 2025 Feb 20;20(2):e0319177. doi: 10.1371/journal.pone.0319177 (PMC11841901; doi:10.1371/journal.pone.0319177)
Supplement: Attachment — Submitted filename: Review of PONE-D-24-52545 Dec 2024.docx [file pone.0319177.s002.docx]

**Review of: Maternal Age and the Rising Incidence of Hypertensive Disorders of Pregnancy: A Comprehensive Analysis of National Claims Data from Japan.**

1. **The study presents the results of original research**

The analysis is based on an extract from the Haikiddo National Insurance Database between 2009 and 2019 – using claims data and the associated codes to make conclusions about the incidence of HDP within the different age groups of pregnancy in the database.

2. **Results reported have not been published elsewhere.**

A brief search did not reveal the same study in Japan published elsewhere.

3. **Experiments, statistics, and other analyses are performed to a high technical standard and are described in sufficient detail.**

Corrections have been made for the challenges of the claims data extraction and the potential for duplication of claims cases (based both on ID numbers and on multiple pregnancies). Adjustment / methodologies have been included and described for both of these challenges. The statistics are largely descriptive with an element of testing for differences of incidences between the different age bands as defined – this has also been handled appropriately. Results presented are those from the defined statistical tests described in the methodology.

4. **Conclusions are presented in an appropriate fashion and are supported by the data.**

Conclusions are fair based on the data presented – as the overall incidence has been increasing over time, the authors surmise that other factors, other than age may also be contributing to the trend (smoking and artificial reproductive technologies are mentioned in the discussion but not speculated about in the conclusion).

5. **The article is presented in an intelligible fashion and is written in standard English.**

Yes

6. **The research meets all applicable standards for the ethics of experimentation and research integrity.**

Retrospective claims database analysis – not experimentation. The authors had their study considered by their institutional ethics board, which waived the ethics approval on the basis of the design and the anonymous data. Author contributions are provided in detail.

Funding provided by the Japan Society for the Promotion of Science (JSPS) KAKENHI, or Grants-in-Aid for Scientific Research.

7. **The article adheres to appropriate reporting guidelines and community standards for data availability.**

Small concern here – the authors have used National Health insurance claims data under the jurisdiction of the Ministry of Health, Labour and Wellness and have stated that this data use has to be within the limitations prescribed by the ministry – this includes limitations on the release of the datasets – this has to be withheld other than the final deliverables. According to the Ministry guidelines, the data also has to be destroyed after the study is completed.

However this is acknowledged in the introductory sections and statement provided in accordance with the guidelines for authors.

The purpose of the review is to provide the editors with an expert opinion regarding the validity and quality of the manuscript under consideration. The review should also supply authors with explicit feedback on how to improve their papers so that they will be acceptable for publication in PLOS ONE. As you write, consider the following points:

8. **What are the main claims of the paper and how significant are they for the discipline?**

The paper focuses on describing known phenomenon e.g. the increase in HDP with age, as well as the focus on the Japanese population. The claims relate to the incidence in the population and how this has grown over time, as well as the raised incidence in older and younger pregnant women, relative to other groups.

The significance in the findings lies in the fact that the age-adjusted incidence of HDP is also increasing – suggesting factors other than age may also be influencing the increase in incidence.

9. **Are the claims properly placed in the context of the previous literature? Have the authors treated the literature fairly?**

Yes and Yes – all the relevant studies I found in Japan have been referenced as well as some key international literature and local and international guidelines on the condition itself.

10. **Do the data and analyses fully support the claims? If not, what other evidence is required?**

Yes – nothing else is required. I felt I would have liked some better researched information on the overall increase in prevalence of HDP and what could be contributing to this – but this would be a different study and design entirely.

11. **PLOS ONE encourages authors to publish detailed protocols and algorithms as supporting information online. Do any particular methods used in the manuscript warrant such treatment? If a protocol is already provided, for example for a randomized controlled trial, are there any important deviations from it? If so, have the authors explained adequately why the deviations occurred?**

No protocol provided – this was a retprospective claims data analysis which used claims data from the insurance database.

12. **If the paper is considered unsuitable for publication in its present form, does the study itself show sufficient potential that the authors should be encouraged to resubmit a revised version?**

Is considered suitable in its current form.

13. **Are original data deposited in appropriate repositories and accession/version numbers provided for genes, proteins, mutants, diseases, etc.?**

No

14. **Does the study conform to any relevant guidelines such as CONSORT, MIAME, QUORUM, STROBE, and the Fort Lauderdale agreement?**

N/A

15. **Are details of the methodology sufficient to allow the experiments to be reproduced?**

Yes – I think the only difficulty may be the reproduction of the same methods in combining ID1 and ID2 numbers from the claims database into a single ID number.

16. **Is any software created by the authors freely available?**

N/A

17. **Is the manuscript well organized and written clearly enough to be accessible to non-specialists?**

Yes – very coherent and clear

18. **Is it your opinion that this manuscript contains an NIH-defined experiment of Dual Use concern?**

No

**Specific notes to Authors:**

Line 187 – the mentioned supplementary table 1 is missing and information not presented. Necessary for review to confirm the estimates.

Line 121 – the Japanese standardised disease codes are not known to all – it may be a good idea to explain these a little better given there are multiple codes applying to the same ICD10 and disease description.

Line 318 – “is older than older gestational age” – should this read “other” or “a factor other than” gestational age?
