# Supplementary material for: Maternal age and the rising incidence of hypertensive disorders of pregnancy: A comprehensive analysis of national claims data from Japan
Source: PLoS One. 2025 Feb 20;20(2):e0319177. doi: 10.1371/journal.pone.0319177 (PMC11841901; doi:10.1371/journal.pone.0319177)
Supplement: Attachment — Submitted filename: Response to reviewers_R1.docx [file pone.0319177.s003.docx]

January 13, 2025

Dr. Preenan Pillay

Academic Editor, *PLOS ONE*

PONE-D-24-52545

“Maternal Age and the Rising Incidence of Hypertensive Disorders of Pregnancy: A Comprehensive Analysis of National Claims Data from Japan”.

Dear Dr. Pillay:

Thank you so much for allowing us to resubmit our manuscript. We thank the editor and reviewers for the careful review of our manuscript. We found their comments constructive and helpful in our revision. The manuscript has been revised and rewritten following the editor's and reviewers' comments. Whole revisions have been written in red font in the manuscript.

Please review our revised manuscript and consider it for acceptance as an original article in *PLOS ONE*.

Thank you for your attention to this letter and the revised version of our manuscript.

Sincerely,

Masayuki Koyama, MD., Ph.D.

Department of Public Health,

Department of Cardiovascular, Renal and Metabolic Medicine

Sapporo Medical University School of Medicine

**To Additional Editor Comments:**

Thank you so much for reviewing and providing useful comments on our manuscript entitled " Maternal Age and the Rising Incidence of Hypertensive Disorders of Pregnancy: A Comprehensive Analysis of National Claims Data from Japan " (Submission ID: PONE-D-24-52545).

The whole manuscript has been revised and rewritten in accordance with your comments.

Our point-by-point responses are presented below.

***1. The statistical analysis applied must be substantiated by supplying the data distribution trends and appropriate justification for the statistical tests applied.***

We appreciate your constructive suggestion. We would like to respond to your comments as follows:
In this study, we aimed to evaluate differences in HDP incidence rates across age groups using robust statistical methods tailored for proportion data. The Wilson score method was employed to calculate 95% confidence intervals for HDP incidence rates, as it is well-suited for proportion data, particularly when sample sizes are intermediate-to-large or proportions are close to 0 or 1. To ensure the validity of the analysis, we assumed that HDP incidence follows a binomial distribution, where the number of successes was defined as HDP cases and the number of trials as births within each age group, with each birth modeled as an independent trial with a constant probability of HDP occurrence. The average number of births from 2011 to 2019 was used for each age group to account for annual fluctuations in birth numbers, providing stable and reliable incidence rate estimates. Differences across age groups were first assessed using the Kruskal-Wallis test to detect overall differences in HDP incidence, a non-parametric method that does not assume specific data distributions. Following this, pairwise comparisons between the reference group (25–29 years) and other age groups were conducted using Z-tests for proportions. The 25–29 age group was chosen as the reference because it had the largest number of births (n = 89,337), making it statistically stable and minimizing sampling bias. To address potential Type I errors due to multiple comparisons, Holm’s method was applied to adjust p-values, ensuring statistical validity. The results of these analyses, including HDP incidence rates and their 95% confidence intervals for each age group, are illustrated in **Figure 4**. These data distribution trends confirmed that the conditions for applying Z-tests were met, as the number of successes (np np) and failures (n (1−p) n (1−p)) in each group satisfied the criterion np ≥ 5 np ≥ 5 and n (1−p) ≥ 5n (1−p) ≥ 5. These rigorous methods ensured the reliability and objectivity of our statistical analysis.


***2. The discussion lacks critical analysis and this must be corrected so that the novelty of the research incomparison to work already done can be justified. There are a number of studies done on the prevalence of HDPs globally. This can be used in the critical discussion, and how does your manuscript address the gaps that previous research has over looked. In addition discuss how claims data can be beneficial to scientific reporting with however limitations regarding the population size as this data only applies to individuals who have access to health insurance. An explanation of this ,major limitation must be given.***

We appreciate your constructive suggestion. In order to reflect your comments, we have examined the draft content and added the following text to the Strengths and Limitations Part (page 20, lines 361- page 22, lines 387).

*This study addresses a significant gap in existing research by evaluating the incidence of hypertensive disorders of pregnancy (HDP) among younger populations, a group that has been largely overlooked in previous studies. Existing research has predominantly focused on older pregnant women or general population-based estimates, often lacking detailed age-stratified analyses. For instance, international studies such as the Global Burden of Disease project have provided valuable insights into overall prevalence trends but have not adequately captured rare conditions like HDP in adolescents. In contrast, Japan’s National Database of Health Insurance Claims and Specific Health Checkups (NDB) allows for comprehensive analyses across a broad range of age groups, including younger individuals, enabling the detailed identification of rare cases and age-specific trends. Leveraging this capability, the present study tracked HDP incidence rates in younger populations over a nine-year period, addressing a critical gap in public health research.*

*Moreover, one of the strengths of the NDB lies in its foundation on Japan’s universal health insurance system, which covers nearly the entire population. This minimizes selection bias while ensuring robust statistical power. The NDB has been widely recognized as a valuable resource for understanding healthcare utilization trends and disease incidence [28–30] [59, 60]. By utilizing real-world clinical data, it provides critical insights into disease management and patient behavior, complementing findings from prospective cohort studies. This study harnessed these features of the NDB to calculate HDP incidence rates in the Hokkaido region, offering a unique perspective on the epidemiology of HDP.*

*Another notable strength of this study is its ability to accurately track the annual trends of HDP over a long period, from 2011 to 2019, prior to the onset of the COVID-19 pandemic. Importantly, the study also includes data covering pregnancies among women aged 19 years or younger, an age group that has been challenging to analyze in prior prospective cohort studies conducted in developed countries. This comprehensive inclusion of younger age groups highlights the distinct contribution of this study to understanding the epidemiology of HDP across diverse demographic categories.*

However, our study has several limitations. First, ・・・・

**To Reviewer 1:**

Thank you so much for reviewing and providing useful comments on our manuscript entitled " Maternal Age and the Rising Incidence of Hypertensive Disorders of Pregnancy: A Comprehensive Analysis of National Claims Data from Japan " (Submission ID: PONE-D-24-52545).

The whole manuscript has been revised and rewritten in accordance with your comments.

Our point-by-point responses are presented below.

15. ***Are details of the methodology sufficient to allow the experiments to be reproduced?***

*Yes – I think the only difficulty may be the reproduction of the same methods in combining ID1 and ID2 numbers from the claims database into a single ID number.*

→ We agree with your comment. **The description of the calculation method for IDT has been strengthened as follows** (page 7, lines 113-120).

Even if the ID1 is different, if the ID2 is the same, it is considered to be the same patient, and even if the ID2 is different, if the ID1 is the same, it is considered to be the same patient (or the ID is reassigned so that it can be considered to be the same).

In practice, the following procedure is used.

STEP 0. All data rows have the ID1, ID2 and IDT columns. Initially, IDT is empty.

STEP 1. Assign the same IDT to the same ID1.

STEP 2. Make sure that the same IDT is used for the same ID2.

STEP 3. Make sure that the same IDT is used for the same ID1.

STEP 4. Repeat steps 2 and 3 until the number of updates reaches zero.

***Areas for correction and improvement:***

- ***Line 187 – the mentioned supplementary table 1 is missing and information not presented. Necessary for review to confirm the estimates.***

We apologize for the lack of attachments in the first draft we submitted. We have added supplementary table 1, which was missing. We would appreciate your confirmation.

- ***Line 121 – the Japanese standardised disease codes are not known to all – it may be a good idea to explain these a little better given there are multiple codes applying to the same ICD10 and disease description.***

We appreciate your constructive suggestion. We have added the following text to the explanation for Table 1 (page 9, lines 138-150).

‘The Japanese standardized disease codes are part of a comprehensive classification system used in Japan to identify and record diseases and conditions. These codes are linked to the International Classification of Diseases, 10th Revision (ICD-10), but include additional details to accommodate Japan's healthcare system and billing practices. While some ICD-10 codes directly map to a single Japanese standardized disease code, others may correspond to multiple codes depending on the context and specific details recorded in medical receipts. For instance, codes for gestational hypertension and preeclampsia may vary based on the level of detail provided in the diagnosis or treatment description. To ensure consistency, this study reviewed and standardized 15 Japanese disease codes explicitly associated with hypertensive disorders in pregnancy (HDP). The mapping process involved careful evaluation of the disease descriptions and their correspondence to ICD-10 codes, as summarized in Table 1. This approach ensures that the selected codes accurately represent HDP cases within the constraints of the NDB.’

- ***Line 318 – “is older than older gestational age” – should this read “other” or “a factor other than” gestational age?***

We apologize for the typographical error in the first submitted draft. We have corrected it and made the aim of the logistic regression analysis clear as follows.

‘is older than older gestational age’

→ “is a factor other than gestational age” (page 19, lines 342).
